# Change in timing of induction protocol in nulliparous women to optimise timing of birth: results from a single centre study

**DOI:** 10.1186/s12884-022-04663-6

**Published:** 2022-04-13

**Authors:** Laura Slade, Georgina Digance, Angela Bradley, Richard Woodman, Rosalie Grivell

**Affiliations:** 1 Flinders Medical Centre, Adelaide, SA Australia; 2 Flinders Health and Medical Research Institute, Flinders University, Adelaide, SA Australia; 3 Flinders University, Adelaide, SA Australia

**Keywords:** Induction of labour, Cervical ripening, Cesarean section, Pregnancy complications, Obesity in pregnancy

## Abstract

**Background:**

Induction of labour (IOL) is a common obstetric intervention. When planning IOL, especially in women at risk for complications at delivery, the aim should be for delivery to occur when senior staff are available to optimise safe care.

**Methods:**

A change in timing of IOL protocol at our institution was introduced in November 2018 aiming to increase births occurring “in-hours” defined as 08:00 to 20:00 h. This retrospective cohort study compares the odds of “in-hours” birth before and after the intervention and the association on birth outcomes. The study compared outcomes during the new IOL pathway period to a historical birth cohort from January to December 2017. Inclusion criteria were nulliparous women undergoing planned IOL at term with a cephalic singleton pregnancy. Logistic regression was used to compare odds of in-hours birth for the 2 periods with adjustment for maternal age at delivery, gestation, more than 2 cervical ripening agents required, undergoing IOL for post-dates pregnancy, mode of birth, whether or not IOL proceeded according to planned protocol and missing values using multiple imputation.

**Results:**

The rate of deliveries occurring in-hours were higher following the intervention; *n* = 118/285 (45.6%) pre-intervention versus *n* = 251/470 (53.4%) post-intervention; adjusted OR = 1.47, 95% CI = 1.07–2.01, *p* = 0.02). The percentage of caesarean sections (CS) occurring in-hours was significantly lower in the pre-intervention group *n* = 71/153 (28.3%) compared with the post intervention group = 35/132(46.4%) (*p* < 0.001)). The rate of CS was higher in the pre intervention *n* = 132/285(46.3%) compared with the post intervention group *n* = 153/470 (32.4%)).

**Conclusions:**

The change in induction procedures was associated with a significantly higher rate of births occurring in-hours and a lower rate of overall of CS. This policy change led to a better pattern of timing of birth for nulliparous women undergoing IOL.

## Introduction

The rate of induction of labour (IOL) in South Australia has been steadily increasing from 29.6% in 2010 [1] to 35.9% in 2017 [2]. Population data reports an increase in the overall rate of pregnancy complications from 34.1% to 43.7% across the same period of time, many of which are recognised indications for induction of labour [3]. These factors contribute to multiple risk factors for adverse maternal and neonatal outcomes, especially in labour and birth [4].

Optimising the outcome of planned birth requires a multidisciplinary team [5]. When planning birth in any woman, but particularly those with risk factors for complications, the aim should be for birth to occur when there is senior presence from obstetrics as well as anaesthetics and neonatalology. Night-time birth has been shown to increase perinatal adverse outcomes including 5-min Apgar score below 7, admission to neonatal intensive care (NICU) and intrapartum or early neonatal mortality [6].

It is important to review protocols and procedures to be able to optimise efficiency and safety in the delivery of obstetric care. The aim of this retrospective cohort study was to review a new IOL pathway was associated with improvements in birth outcomes for women undergoing planned delivery.

## Materials and methods

In November 2018, a change in timing of induction procedure was introduced at a large tertiary maternity unit with both maternal and neonatal intensive care facilities in Adelaide, South Australia. This study compares birth outcomes for women undergoing IOL in the new pathway (post-intervention group), with those for the old pathway (pre-intervention group). All nulliparous women undergoing induction of labour (IOL) with a cephalic term singleton who required cervical ripening were identified from the hospital administrative database. IOLs conducted more urgently due to a clinical concern were also included if the timing was in accordance with the protocolised admission times. All other admission times and those women who did not require cervical ripening or those with ruptured membranes were excluded.

The procedure change was initiated as a quality assurance project because audit review identified that a significant proportion of deliveries, particularly caesarean sections, were occurring late in the evening or overnight. Many of these births involved complexities such as obesity or full dilatation. The aim of adjusting the procedure was that more births would occur within the obstetric medical staff day-shift; 08:00 h to 20:00 h, defined as “in-hours”. During this time, both the total number of staff and in particular senior staff, is greater.

**Fig. 1:**
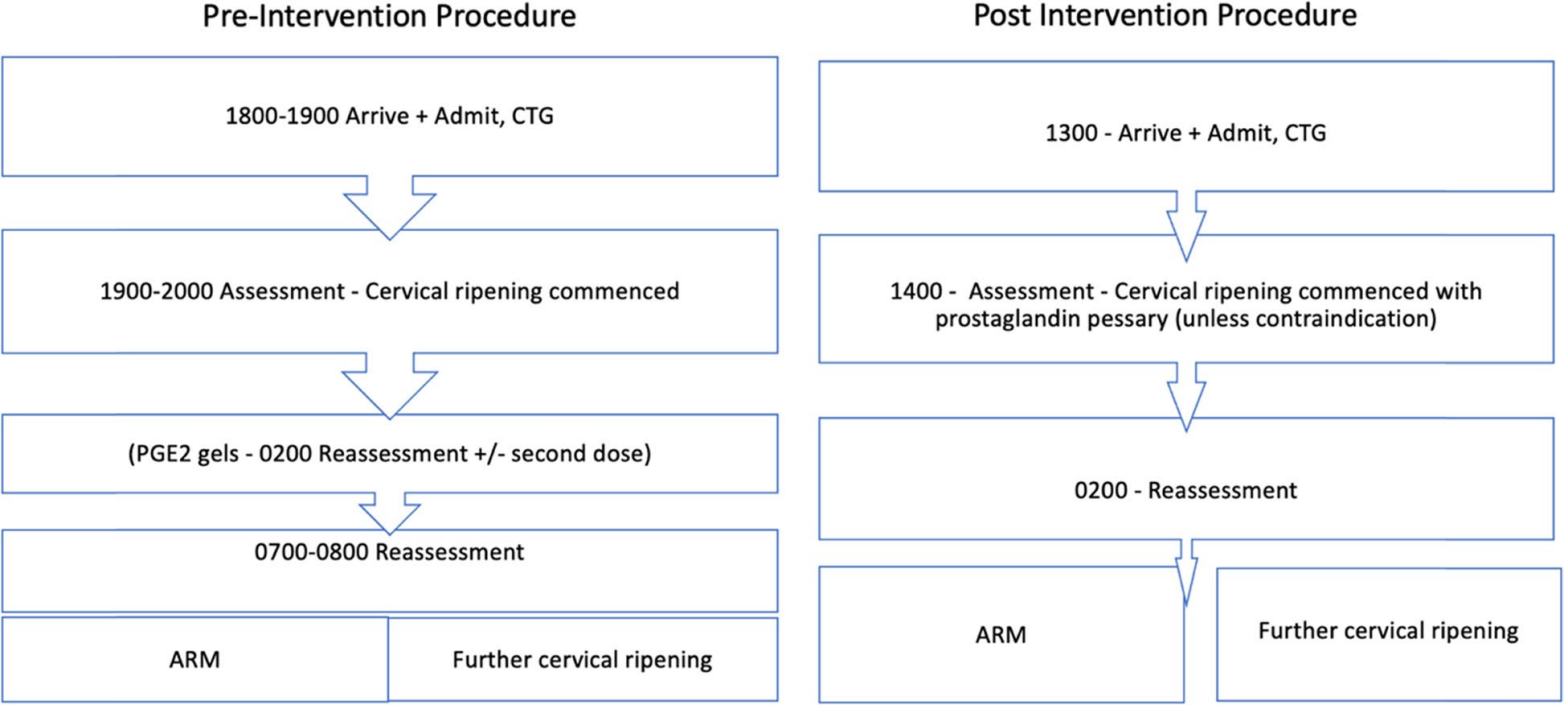
Flow chart of pre-intervention vs post-intervention induction of labour procedure. CTG – cardiotocogram, ARM – artificial rupture of membranes, PGE2 gels – prostaglandin gel for cervical ripening

To create the new protocol, modelling of induction timing was performed based on the from the pre-intervention period (January to December 2017), which included all nulliparous women with a term cephalic singleton undergoing IOL requiring cervical ripening with an admission from 18:00 to 20:00. At this time, the pathway had involved admission at 18:00 h, cervical ripening with either prostaglandin gels or a cervical ripening balloon with reassessment for ARM aimed at 08:00 h (Fig. 1). If the cervix remained unfavourable for ARM at that time, defined by a Modified Bishop’s score of less than 7, reassessment was undertaken to plan continuing ripening. During this period, ripening was undertaken with either prostaglandin gel or a balloon catheter or a sequential combination if required.

The post intervention protocol involved admission at 13:00 h with cervical ripening with a dinoprostone pessary inserted at 14:00 (Fig. 1). This was left for 12 h and then artificial rupture of membranes (ARM) performed if the cervix was favourable at 02:00 h. If further cervical ripening was required as deemed by a Modified Bishop’s score of less than 7, the pessary was repositioned and further reassessment was undertaken another 6 h later at 08:00 h. If the Modified Bishop’s score remained less than 7 and hence unfavourable for ARM at that time, clinician review was undertaken and a discussion about further ripening with dinoprostone, prostaglandin gel or balloon catheter was had with the woman. Further ripening depended on maternal and clinician preference.

Data were collected through the hand written birth record and then individually confirmed on review of the electronic maternity database. Demographic, obstetric and delivery information was collected for each case and analysed pooling all deliveries to assess the overall pattern of delivery timing, stratified by mode of delivery. All listed indications for IOL were recorded with no weighting given to different indications and if more than one was listed. The Modified Bishop’s score was that recorded at admission for IOL and was recorded by the midwife inserting the cervical ripening agent at the time of admission.

The primary outcome was birth in-hours defined by birth between 08:00 and 20:00 to correspond with the highest levels of staffing throughout the day. The secondary outcomes were the mode of birth and the rate of caesarean section, spontaneous birth and assisted vaginal birth in each group.

The time between the pre and post intervention periods reflects the time taken to collect and analyse the data required, and refine the process through the necessary channels within the Obstetric Unit at the hospital. A post-hoc power calculation demonstrated that the overall sample size of *n* = 765 provided 80% power to detect an odds ratio of 1.5 to for the post versus pre-intervention periods, assuming an underlying in-hours delivery rate of 40%, a type-1 error rate of alpha = 0.05 and 62% of the patient cohort within the post-intervention period.

### Statistical analysis

Patient characteristics were described according to the pre and post intervention periods using the mean and standard deviation for normally distributed continuous variables and median (inter-quartile range) for non-normally distributed variables. Categorical variables were described using frequency counts and percentages. Differences in patient characteristics between the two time periods were assessed using independent t-tests and chi-squared tests of association.

We used univariate and multivariate logistic regression to assess the association between the pre and post intervention periods and in-hour delivery. To account for potential mediating factors, we adjusted for age at delivery and gestation (Model 2) and then further adjusted for the need for 2 or more cervical ripening agents, induction indication as post dates pregnancy and mode of birth, as well as whether or not the induction proceeded according to the planned procedure assessed by whether or not artificial rupture of membranes was performed within 2 hours of the target time (Model 3). Due to approximately 22% of patients having at least one of these covariate values missing, and therefore being lost from the multivariate analysis, we also performed multiple imputation using chained equations to obtain 5 sets of data, each with complete covariate values for all observations (Model 4). These 5 datasets were used in a repeat of the multivariate regression analysis using the appropriate Stata regression commands for multiply imputed datasets. Variables included in the imputation process included all the covariates used in the regression analyses. A 2-sided Type 1 error rate of alpha = 0.05 was used for all significance testing. Descriptive statistics, regression analysis and multiple imputation was performed using Stata version 1.0 (StatCorp, USA).

The project was submitted to the Southern Adelaide Local Health Network Office for Research but as an audit project the study met criteria for exemption from this review according to local institutional policy with reference number 251.20, therefore no formal ethics approval was required. As the protocol was introduced, regular reviews were conducted at monthly unit meetings with formalised reviews at 6 monthly intervals.

## Results

A total of *n* = 755 patients were included in the analysis. This included 285 women from the historical cohort from January to December 2017, and 470 women who underwent IOL with the new protocol between November 2018 and June 2020. Complete data for all variables included in the analysis were available for in-hours delivery, intervention period, gestation, induction indication, and delivery type. However, there were missing values for delivery BMI (*n* = 69), age at delivery (*n* = 81), ripening type (*n* = 4), more than 2 agents required for cervical ripening (*n* = 4) and ARM in target time (*n* = 25). In total, *n* = 166 (22.0%) had at least one missing value for these variables.

There was a significant difference between the percentage of IOLs performed at 41 or more completed weeks gestation from 26.3% of the inductions in the pre-intervention group to 17% of overall IOLs in the post intervention group (*p* < 0.01). In accordance with this, the percentage of women undergoing IOL for postdates pregnancy was also significantly lower in the post intervention group (*p* = 0.02) (Table 1). There was no difference in Modified Bishop’s score on admission between the groups. Other demographic factors were not significantly different. The preferred cervical ripening agent used at changed between the time periods within the institution, all women in the post intervention group commencing ripening with a prostaglandin pessary.

**Table 1:** Demographics across delivery periods

	Pre-intervention *n* = 285	Post intervention *n* = 470	*p* value^a^
Age at delivery, mean (SD)	29.06 (5.04)	29.13 (5.17)	0.87
BMI at delivery, median (IQR)	30.9 (27.0, 35.5)	31.55 (27.6, 37.8)	0.10
Gestation (weeks), mean (SD)	39.2 (1.5)	39.1 (1.3)	0.26
Induction indication (n, (%)):δ			
- Post-dates	- 77 (27.0)	- 93 (19.8)	- 0.02*
- Diabetes mellitus (GDM, T1DM, T2DM)	- 79 (27.7)	- 128 (27.1)	- 0.89
- HDP	- 48 (16.8)	- 56 (11.9)	- 0.06
- IUGR	- 23 (8.1)	- 42 (8.9)	- 0.68
- Reduced fetal movements	- 15 (5.2)	- 47 (10.1)	- < 0.01
- LGA	- 30 (10.5)	- 69 (14.6)	- < 0.01
Modified Bishop’s score on admission	3 (2–4)	3 (2—4)	
Cervical ripening agent			
- Prostaglandin gel	76 (26.7%)	69 (14.7%)	< 0.01
- Cervical ripening balloon	185 (64.6%)	18 (3.9%)	< 0.01
- Prostaglandin pessary	-	472 (100%)	-
- Multiple agents	25 (8.8%)	82 (17.5%)	< 0.01

^a^Using independent t-test or chi-squared test as appropriate

*T1DM* type 1 diabetes mellitus, *T2DM* type 2 diabetes mellitus, *GDM* gestational diabetes mellitus, *HDP* hypertensive disorders of pregnancy (including gestational hypertension and preeclampsia), *IUGR* intrauterine growth restriction, *LGA* large for gestational age (> 90^th^ centile)

δ—all listed indication for induction each recorded separately

There was a significant difference overall in the percentage of deliveries in-hours between the two groups; *n* = 118/285 (45.6%) in the pre-intervention group compared with *n* = 251/470 (53.4%) for the post intervention group (Table 2). There was also a significant difference in the rate of CS with 46.3% in the pre-intervention group compared with 32.5% of women in the post intervention group (Table 2). The proportion of CS performed in-hours was significantly different with 28.0% of CS occurring in hours in the pre-intervention group compared with 46.4% with the post intervention group (Table 2).

**Table 2:** Descriptive statistics for mothers and their birth characteristics by intervention period

	Pre-intervention		Post-intervention		*p*-value^^^
Mode of birth	Total n	In hours n (%)	Total n (%)	In hours n (%)	
SVB	101	70 (69.3)	180	98 (54.4)	0.02
AVB	52	23 (44.0)	137	82 (60.0)	0.01
CS	132	35 (26.5)	153	71 (46.4)	< 0.01
Total	285	118 (41.4)	470	251 (53.4)	< 0.01

^^^Using independent t-test or chi-squared test as appropriate

*SVB* spontaneous vaginal birth, *AVB* assisted vaginal birth, *CS* caesarean section

In logistic regression analysis, the odds of in hours births was higher in the post-intervention group in both univariate (OR = 1.62, 05% CI = 1.20, 2.18; *p* < 0.01) and in the fully adjusted model with multiple imputation (fully OR = 1.47; 95% CI = 1.07–2.01, *p* = 0.02) (Table 3).

**Table 3:** Association between Post versus Pre-intervention periods and In-hours delivery

	In-hours birth n (%)		Model 1 (*n* = 755) Odds ratio (95% CI) (*p*-value)	Model 2 (*n* = 674) Odds ratio (95% CI) (*p*-value)	Model 3 (*n* = 589) Odds ratio (95% CI) (*p*-value)	Model 4 (*n* = 749) Odds ratio (95% CI) (*p*-value)
	No	Yes				
Period:						
Pre-intervention	167 (58.6)	118 (41.4)	1.00 (Ref)	1.00 (Ref)	1.00 (Ref)	1.00 (Ref)
Post-intervention	219 (46.6)	251 (53.4)	1.62 (1.20, 2.18) *P* < 0.01	1.56 (1.15, 2.12) *P* < 0.01	1.38 (0.97, 1.97) *P* = 0.07	1.47 (1.07, 2.01) *P* = 0.02

Model 1: Unadjusted

Model 2: Adjusted for age at delivery and gestation

Model 3: Adjusted for age at delivery, gestation, ≥ 2 ripening factors, post-date, mode of birth, and ARM in target-time

Model 4: Same as model 3 and using multiple imputation (*n* = 5 imputed datasets)

## Discussion

Studies comparing protocols for induction of labour are scarce. The Cochrane review of morning versus evening induction of labour showed no difference in maternal or neonatal outcomes or rates of caesarean Sect [7]. A recent study from Queensland, Australia demonstrated that adjustments in induction protocols can increase the proportion of in-hours deliveries defined in their study as 07:00 to 19:00. Beckmann and colleagues adjusted admission times from 19:00 h to 11:00 for both nulliparous and multiparous women. This led to a 14.7% increase in the rate of in-hours births and a decrease in the time of delay to induction [8].

In our initial review of induction procedures and deliveries, the multiparous women delivering at our institution already had relatively high rates of in-hours delivery and low rates of caesarean section prior to the introduction of the intervention. No changes were made for multiparous women in our intervention adjustment. This resulted in a change from all women arriving at the same time each evening, to women in the post intervention group being due for assessment and routine cares at different times overnight. The aim of this was that planned initial workload would occur at dispersed intervals, whilst births were more likely to occur during in-hours periods.

There is evidence that unscheduled CS performed out-of-hours is associated with higher rates of adverse maternal and neonatal outcomes. A large retrospective study from Turkey compared morning, evening and night time unplanned caesarean sections and demonstrated increased rates of a maternal morbidity composite that included endometritis, wound infection and postpartum haemorrhage with women who were delivered on the night shift between 23:00 and 07:00 h [9]. Perinatal morbidity or mortality was also increased with evening or night birth (defined as 18:00 to 08:00 h) in a retrospective review from the Netherlands in which births most at risk were those involving induction or augmentation of labour or delivery by emergency caesarean section. In this review, infants born in those same out-of-hour’s time periods after spontaneous labour were not at increased risk of adverse perinatal outcomes [10].

Optimising outcomes for mothers and infants has many challenges. The unpredictability of patterns in workload and balancing planned and unplanned clinical activity are particular difficulties. It seems sensible that the planned workload of maternity care should be aimed to occur when staffing levels are at the maximum level. This decision is however compounded by the unpredictability of the outcome of IOL in nulliparous women and the broad range of times from induction to delivery [11].

Audit outcomes for nulliparous women undergoing IOL at our institution identified a pattern of delivery occurring in the late evening and overnight. The aim of adjusting the procedure of IOL was beneficial to improve the proportion of complex deliveries occurring in hours during times of peak staffing. We had also hoped to disperse the planned daily workload more evenly across the day meaning less occasions where multiple women were requiring reassessment at the same time. This study did not have the capacity to assess the impact on staffing or workload throughout the day.

Across the post intervention period, 17% of women required ongoing ripening after the dinoprostone pessary. This was either after 12, 18 or 24 h of dinoprostone and varied with whether or not there had been a partial response and also on clinician preference. Despite this, the pattern of timing of birth was still more favourable than the pre-intervention period. This group highlights a potential opportunity for further adjustment of protocol and intervention to optimise the timing of IOL procedures. A more consistent prediction of the duration of cervical ripening required confers better counselling of women about the expectations of the induction duration as well as optimising work flow and staffing arrangements.

The lower rates of CS in the post intervention group is likely multifactorial, however difference remained significant after adjustment for multiple factors known to be associated with a higher CS rate. In the pre-intervention period, the rate of caesarean section was 46.3%. One significant contributor to this high rate is that our institution acts as the referral centre for multiple regional units and overall cares for a higher proportion of obese women and high-risk pregnancies because of this. The CS rate in the post intervention group was 32.6% which is similar to other tertiary centres within Australia when looking at nulliparous singleton cephalic pregnancies [12]. The aim of this pathway was that having an obstetric consultant present on labour ward would assist junior staff with decision making and labour management, promoting safe vaginal delivery. Although a difficult aspect to specifically quantify, the increase in the percentage of instrumental deliveries would suggest that senior decision making has supported some of the change from caesarean section to vaginal delivery in these women. This is consistent with a large meta-analysis from the United Kingdom which found lower likelihood of CS with increased consultant presence [13].

The strengths of this project include the inclusion of a broad range of women with varying levels of complexity and complication to their pregnancy and birth. However, we adjusted for a large number of potential mediating factors and also imputed values for missing data to ensure a lack of unbiased estimates of the effect of the intervention. A limitation of this project was the change in cervical ripening agent at the same time as changing the IOL procedures. Another major limitation of this study is the lack of assessment of the impact of these changes on women’s experience of IOL. In addition, the study was observational in nature rather than experimental and therefore the associations observed cannot be considered as causal.

Adjusting the timing of routine procedures for induction of labour improved the proportion of births occurring at times with peak staff available. The optimisation of staffing and particularly the timing of presence of senior staff may have also contributed to a lower caesarean section rate in the post intervention group.

## Data Availability

The datasets analysed during the current study are available from the corresponding author on reasonable request.
